# Effect of human umbilical cord blood stem cell transplantation on oval cell response in 2-AAF/CCL4 liver injury model: experimental immunohistochemical study

**DOI:** 10.1186/s41232-017-0035-8

**Published:** 2017-02-06

**Authors:** Hussein Abdellatif, Gamal Shiha, Dalia M. Saleh, Huda Eltahry, Kamal G. Botros

**Affiliations:** 1 Anatomy and Embryology Department, Faculty of Medicine, University of Mansoura, Mansoura, Egypt; 2 Internal Medicine Department, Faculty of Medicine, University of Mansoura, Mansoura, Egypt; 3 Egyptian Liver Research Institute and Hospital (ELRIAH), Mansoura, Egypt

**Keywords:** Liver, Chronic injury, Oval cells, OV-6, CD34, hUCB

## Abstract

**Background:**

Oval cells, specific liver progenitors, are activated in response to injury. The human umbilical cord blood (hUCB) is a possible source of transplantable hepatic progenitors and can be used in cases of severe liver injury. We detected the effect of hUCB stem cell transplantation on natural response of oval cells to injury.

**Methods:**

Twenty-four female albino rats were randomly divided into three groups: (A) control, (B) liver injury with hepatocyte block, and (C) hUCB transplanted group. Hepatocyte block was performed by administration of 2-acetylaminofluorene (2-AAF) for 12 days. CCL4 was administrated at day 5 from experiment start. Animals were sacrificed at 9 days post CCL4 administration, and samples were collected for biochemical and histopathological analysis. Oval cell response to injury was evaluated by the percentage of oval cells in the liver tissue and frequency of cells incorporated into new ducts.

**Results:**

Immunohistochemical analysis of oval cell response to injury was performed. There was significant deviation in the hUCB-transplanted (4.9 ± 1.4) and liver injury groups (2.4 ± 0.9) as compared to control (0.89 ± 0.4) 9 days post injury. Detection of oval cell response was dependant on OV-6 immunoreactivity. For mere localization of cells with human origin, CD34 antihuman immunoreactivity was performed. There was no significant difference in endogenous OV-6 immunoreactivity following stem cell transplantation as compared to the liver injury group.

**Conclusions:**

In vivo transplantation of cord blood stem cells (hUCB) does not interfere with natural oval cell response to liver injury.

## Background

Liver stem cell participation in recovery of the severely injured liver have been extensively described. Nevertheless, the exact location of such stem cells is still controversial although bile ducts have been implicated [1]. In experimental carcinogenesis proliferation of the so-called oval cells has been described. These cells are small with oval nuclei that reside in the periphery of the portal tracts in rat models of hepatocarcinogenesis and injury. Oval cells are bi-potent progenitors capable of differentiation into both hepatocytes and cholangiocytes [2]. Their precise nature remains unclear with debate as to whether they are derived from a postulated stem cell or are themselves facultative stem cells. An enormous range of markers has been used for oval cell identification, such as albumin, alpha fetal protein (AFP), epithelial cell adhesion molecule (EpCAM), cytokeratin 7 (CK7), OV-6, and cytokeratin 19 (CK19) [3, 4]. Human umbilical cord blood may be a possible source of transplantable hepatic progenitors. Newsome and his colleagues demonstrated that human umbilical cord blood (hUCB) stem cells could differentiate into hepatocytes after transplantation into immunodeficient mice [5]. Besides, some antigens traditionally associated with hematopoietic cells (c-kit, flt-3, CD34) can be expressed by oval cells/hepatic progenitor cells (HPCs) as well, leading to the notion that at least some hepatic oval cells are directly derived from a precursor of hematopoietic origin [6]. The underlying mechanism of stem cell plasticity and transdifferentiation into mature hepatocytes is of considerable interest. Exploring the therapeutic potential and role of oval cells in the process of natural repair of the liver is of great value and carries much hope in the future for the treatment of liver disease. Studying cord blood stem cells as a source of transplantable liver progenitors, and whether this may interfere with the natural response of oval cells to injury, to our knowledge is still unclear. Thus the aim of the current work was directed to study the effect of hUCB stem cell transplantation on the natural response of oval cells to liver injury.

## Methods

### Collection of human umbilical cord blood

Human umbilical cord blood was collected from full-term pregnant women (Department of Obstetrics and Gynecology, Mansoura University) just before placental separation in normal vaginal delivery after taking informative consent. Participants were considered eligible for the study according to the following exclusion (no family history of gene-based disorders or maternal fever during labor) and inclusion (delivery occurring less than 24 h after rupture of membranes) criteria.

### Separation of the mononuclear cells

Human umbilical cord blood samples were diluted in Dulbecco’s modified Eagle’s medium (DMEM) (1:1) supplemented with 10% fetal bovine serum (FBS). Low-density mononuclear cells were collected after centrifugation at 800×*g* for 20 min in Ficoll density gradient (Histopaque, 1.077 g ml^−1^) following the manufacturer’s instructions. Mononuclear hematopoietic cells were obtained from the interphase and washed twice with sterilized PBS. Pellets were re-suspended in lysis buffer (150 mM NH_4_ Cl, 1 mM KHCO_3_, 0.1 mM Na-EDTA, pH 7.4) and incubated for 5 min at 4 °C to deplete erythrocytes. After washing once with PBS, pellets were again re-suspended. Cell viability, determined by the trypan blue dye exclusion method, was 97.40 ± 0.43%. The total average number of viable cells isolated from one umbilical cord was 8 × 10^7^ [7].

### Animal preparation

Twenty-four adult female albino rats (Cux1: HEL1) 12 weeks of age, weighing 200–250 g. Rats were bred and maintained in an air-conditioned animal house (Medical Experimental Research Center, MERC, Mansoura University) (under controlled temperature 25 ± 2 °C) with specific pathogen-free environment and were subjected to a 12:12-h daylight/darkness cycle and allowed free access to rat chow and water. The principles of laboratory animal care were fulfilled in all experimental protocols and were approved by the ethics committee of animal research in MERC.

### Animal groups

Rats were randomly divided into the following groups:

- *Control group* (*n* = 8). The rats received corn oil (10 ml kg^−1^ B.W.) orally throughout the whole experiment.
- *Liver injury and hepatocyte block group* (*n* = 8). The rats received acetylaminofluorene (2-AAF) (A7015, Sigma-Aldrich) to inhibit hepatocyte proliferation. The drug was administered in a dose of (10 mg kg^−1^ B.W.) as daily oral gavage for 12 days. 2-AAF was dissolved in a small volume of dimethyl sulfoxide (DMSO; Sigma cat: D8418) and suspended in corn oil to a final concentration of 2 mg ml^−1^. On day 5 from the start of the experiment, rats received carbon tetrachloride (CCL4) in a dose of 0.6 ml kg^−1^ B.W. dissolved in corn oil (1:1) to induce differentiation of oval cells.
- *Stem cell transplantation group* (*n* = 8). The rats were subjected to 2-AAF/CCL4 liver injury protocol as described before; in addition, they also received human umbilical cord blood stem cells (mononuclear cells, MNCs) as a source of exogenous stem cells to the liver. This group received hUCB MNCs (8 × 10^6^) in 0.3 ml media injection (DMEM) over 3 min through the portal vein on the day following CCL4 administration. Rats in different groups were sacrificed at day 9 post CCL4 injury.

### Biochemical analysis

Heparinized blood samples were obtained from all animals at planned time intervals. Aspartate transaminase (AST) and alanine transaminase (ALT) activities and serum albumin and total bilirubin levels in the plasma were determined using commercially available kits (Sigma Chemical Co. kit nos. 58 and 59).

### Specimens’ collection

The livers were harvested following in situ perfusion through the portal vein using Ca^++^-free Hank’s balanced salt solution (HBSS) (pH 7.4). The initial flow rate was 15–20 ml min^−1^ with the perfusate exiting through the inferior vena cava. The livers were divided into pieces and were used for histopathological studies.

### Staining

Small pieces of liver were collected and fixed overnight in 10% neutral-buffered formalin. The fixed liver tissue was then dehydrated in ascending grades of alcohol and then cleaned by xylene then embedded in paraffin. Five-micrometer-thick sections were mounted on clean glass slides. Sections were stained with hematoxylin-eosin (HE) for histopathological changes, antihuman/rat OV-6 antibody (R&D Systems, MAB2020) for the detection of oval cells that may originate from human or rat source, and antihuman CD34 monoclonal antibody (Thermo Scientific, MAI-21937) directed only for the detection of cells that originate from a human source (hUCB-derived cells).

### OV-6 and CD34 immunohistochemistry

Tissue sections were rehydrated in decreasing concentrations of ethanol, and endogenous peroxidase activity was blocked with 0.5% hydrogen peroxide in methanol. The tissue was microwaved to boiling for 20 min in 0.1 mol l^−1^ of Tris EDTA pH 9.0 for antigen retrieval. The antibody was diluted in phosphate-buffered saline plus 0.1% nonfat dried milk and applied at 4 °C for 12 h. Primary antibody dilution was 1:10 for OV-6 and 1:80 for CD34. The tissue was incubated with biotinylated anti-mouse secondary antibody in a species-specific manner (R&D Systems, MAB002). The label was peroxidase-conjugated streptavidin. Color development was performed with diaminobenzidine peroxidase substrate (D-4293, Sigma Chemical Co.). Finally, sections were counterstained for 2 min with hematoxylin, dehydrated through graded alcohols, and mounted under glass cover slips.

### Image analysis

Analysis of oval cell response was done using an Olympus microscope at a total magnification of 400, captured by digital camera (Olympus LC20) and using the image analyzer software (LC micron). The image analyzer was calibrated at first to convert the measurement units (pixels) produced by the image analyzer program into actual micrometer units. Oval cells were quantified by counting cells positive for OV-6 antigen, relative to total hepatocyte numbers, in 10–15 randomly selected fields of the portal tracts in a blinded way at 400× magnification. The cell count is then expressed as a percentage in relation to the hepatocyte number.

The oval cell response and the frequency of oval cell incorporation into new ducts were scored as (1) oval cells constituting 10% of the field and only 10% of oval cells were incorporated into new ducts, (2) oval cells constituting 20% of the field and only 20% of oval cells were incorporated into new ducts, (3) oval cells constituting 30% of the field and 30% of oval cells were incorporated into new ducts, (4) oval cells constituting 40% of the field and 40% of oval cells were incorporated into new ducts, and (5) oval cells constituting 50% of the field and 50% of oval cells were incorporated into new ducts. Average cell percentages were then pooled for each experimental group, and the overall mean and standard deviation were determined, and then the overall response was scored according to the previously described score.

## Statistical analysis

As the oval cell reaction is not uniform around all the portal tracts over their complete size range and the reaction varies between different animals at different time intervals, the coefficient of variation (CV) was calculated and it was statistically significant for all groups. Data are expressed as mean ± SD. Multiple comparisons were done using SPSS 15.0 computer software. Significance of results was considered at *P* value <0.05.

## Results

### Biochemical analysis

Nine days post CCL4 injection, ALT and AST levels were estimated in the blood of all experimental groups and were significantly elevated in the liver injury (114 ± 37.5, 265 ± 127.3) and stem cell transplantation groups (132.8 ± 21.1, 162.5 ± 34.8) as compared to the control group (42.6 ± 2.8, 44.3 ± 3.5), respectively. Data are expressed as mean ± SD. Pairwise comparison between sample means reveals significant difference between groups. Data are considered significant at *P* value <0.05. Serum albumin and bilirubin levels were also estimated in all groups, and no significant difference was found in the liver injury (3.9 ± 0.2, 0.31 ± 0.16) or stem cell transplantation group (3.95 ± 0.13, 0.28 ± 0.11) as compared to the control (3.9 ± 0.3, 0.31 ± 0.18), respectively, as indicated by *P* value >0.05 (Table 1).

**Table 1:** Plasma levels of ALT, AST, albumin, and bilirubin in rats

	Group A control	Group B liver injury group	Group C (2AAF/CCL4 + stem cell transplantation)
At 9 days post CCL4 injury			
ALT	42.6 ± 2.8	114 ± 37.5*	132.8 ± 21.1*
AST	44.3 ± 3.5	265 ± 187.3*	162.5 ± 34.8*
Albumin	3.9 ± 0.3	3.9 ± 0.2	3.95 ± 0.13
Bilirubin	0.31 ± 0.18	0.31 ± 0.16	0.28 ± 0.11

Values are expressed as means ± SD

*Significant deviation from the control group as indicated by *P* < 0.05

### Changes in oval cell response in hematoxylin and eosin stained liver sections

**Fig. 1:**
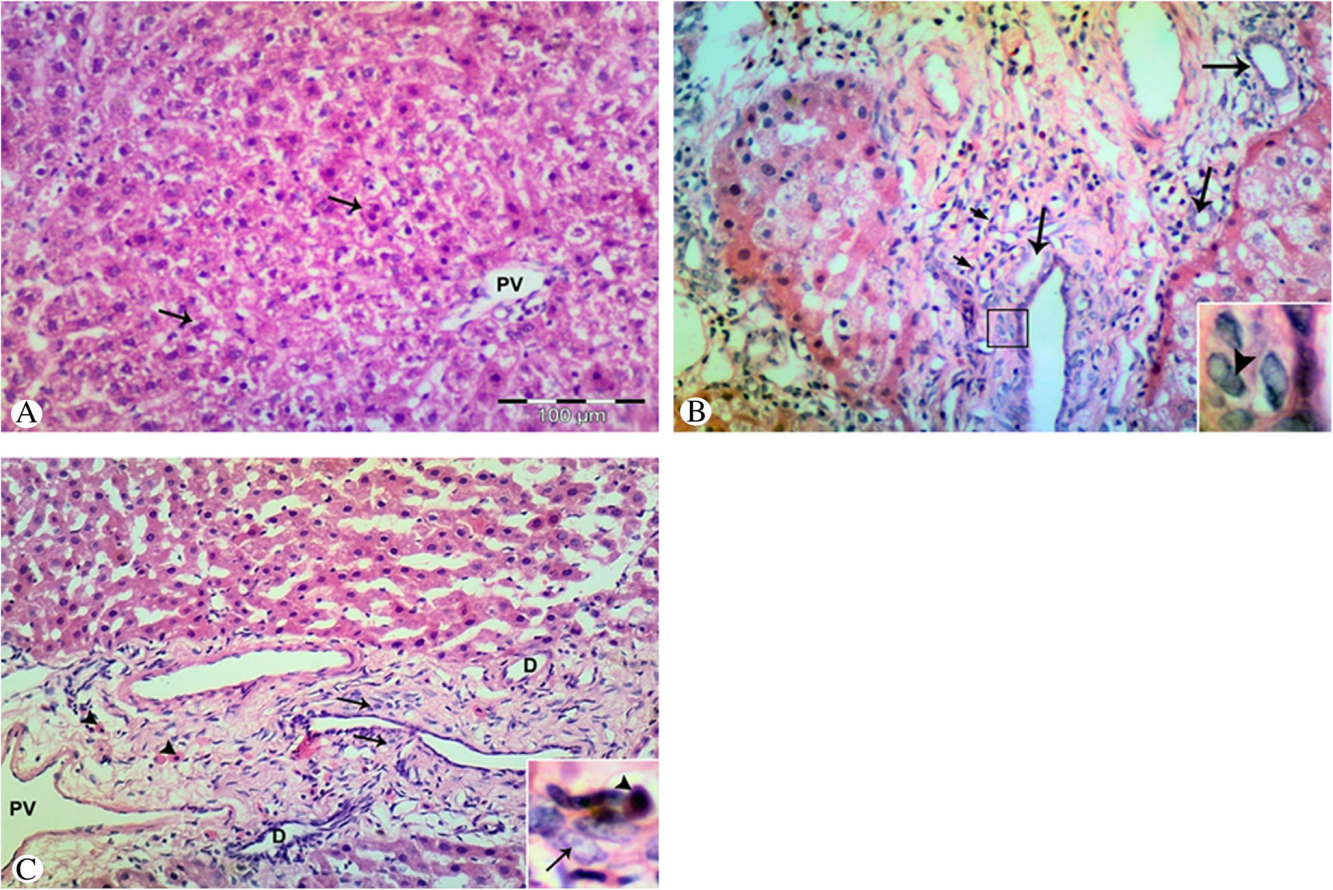
Photomicrograph of liver tissue in the control (**a**), liver injury (**b**), and stem cell transplantation groups (**c**). **a** Normal liver architecture, some hepatocytes, are binucleated (*arrows*). No signs of oval cell response around the portal vein (*PV*). **b** Oval cell response with ductular reaction (*arrows*) and small hepatocyte-like cells (*short arrows*). **c** Increased oval cell response (*arrows*) with ductular reaction (*D*) around portal vein (*PV*). Small hepatocyte-like cells (*arrowheads*). Inset: high magnification showing oval cells (*arrowhead*) with scanty basophilic cytoplasm (*arrow*) and high nucleo-cytoplasmic ratio (**b**, **c**). H&E, 100×; Inset, 400×

No evidence of oval cells was observed in the control group (Fig. 1a). In the 2-AAF/CCL4 group, oval cells appeared as small oval-shaped cells proliferating in files between hepatocytes in the periportal regions or extending beyond the periportal region. These cells had scanty basophilic cytoplasm, dark staining nucleus with a large nucleo-cytoplasmic ratio (Fig. 1, inset). The cells radiate from the periportal region in the form of individual cells or ductular-like structures (Fig. 1b, arrowheads). Some cells also appeared as individual oval-shaped cells with hepatocyte phenotype (small hepatocyte-like cells) (Fig. 1b, short arrows). In the stem cell transplantation group, there was evident increase in oval cells surrounding the portal tract or emanating from it and invading the liver parenchyma extending far beyond the periportal region (Fig. 1c, arrows). Pairwise comparison between groups reveals a highly significant result in the stem cell transplantation group (4.8 ± 1.5, 4.5 ± 1.4) than in the liver injury (3.5 ± 1.4, 3.3 ± 1.5) and control groups regarding oval cell score and duct formation score, respectively (*P* value <0.05).

### Changes in oval cell response detected by immunoreactivity (IHC methods)

**Fig. 2:**
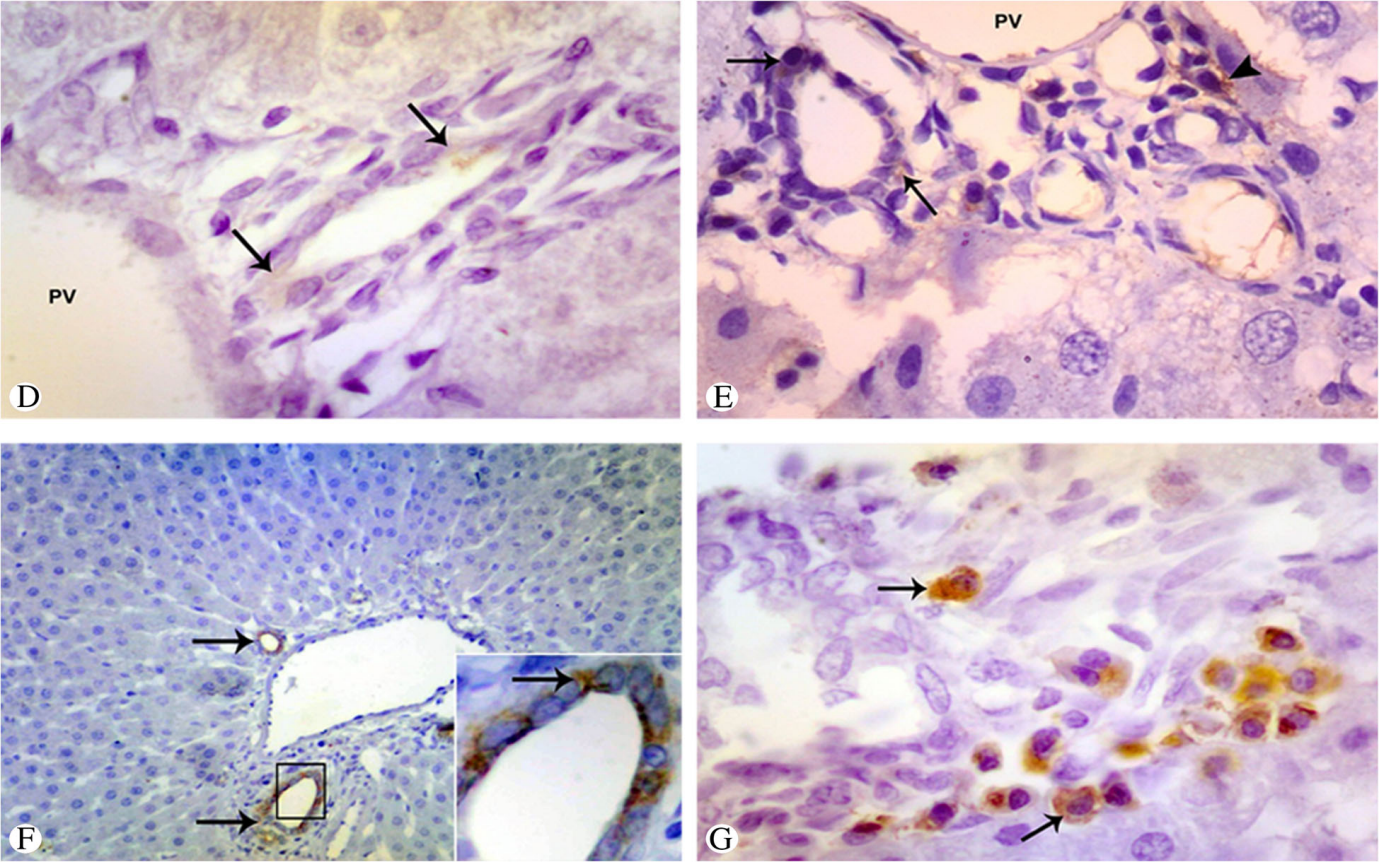
Photomicrograph of liver tissue in the control (**d**), liver injury (**e**), and stem cell transplantation groups (**f**, **g**). **d** Mild immunoreactivity to OV-6 antibody limited to intraportal bile duct and terminal duct cells (*arrows*) surrounding the portal vein (*PV*). **e** OV-6 immunoreactivity (cytoplasmic reaction) with oval cells forming more reactive ductules (*arrows*) or appearing as small hepatocyte-like cells (*arrowhead*). The cells are surrounding the portal vein (*PV*) or emanating from it and invading liver parenchyma. **f** Increased OV-6 immunoreactivity with oval cells forming more reactive ductules (*arrows*) around the portal vein. Inset: Positive oval cells in the reactive ductules (*arrow*), 400×. **g** CD34 immunoreactivity of the human oval cells (*arrows*) scattered and invading the liver parenchyma. Immunoperoxidase stain, 400×

**Fig. 3:**
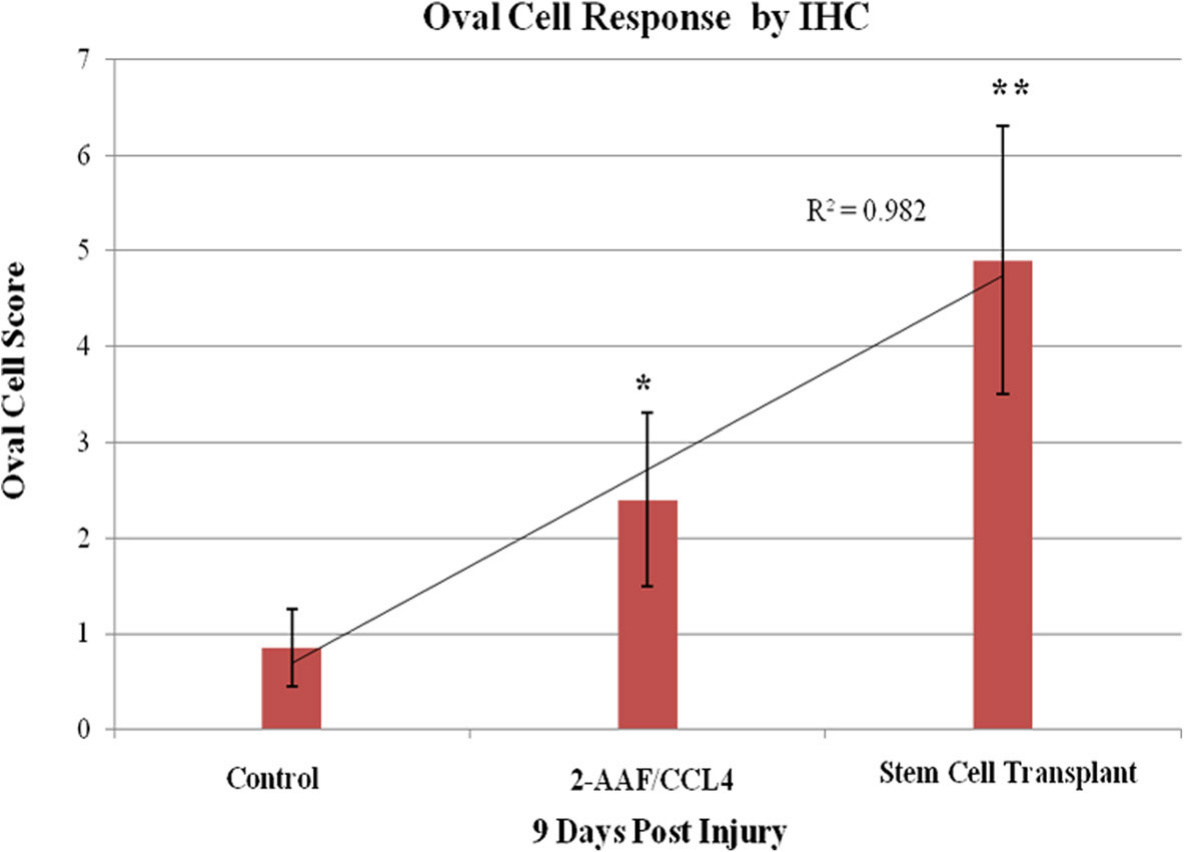
Oval cell response detected by OV-6 immunoreactivity showing highly significant increase in the stem cell transplantation group (*double asterisk*) as compared to the liver injury (*asterisk*) and control groups (*P* value <0.01). Data are expressed as mean ± SD

In the control group, mild immunoreactivity to OV-6 antibody was only limited to the intraportal bile duct and terminal duct cells located at the Canals of Hering (Fig. 2d, arrows). No CD34 positive cells were present in the stained liver samples (no cross-reactivity or evidence of human-derived cells). Oval cell response was 0.85 ± 0.4 and 0.78 ± 0.3 regarding the oval cell score and duct formation score, respectively. In the liver injury and hepatocyte block group, marked increase in OV-6 immunoreactivity (2.4 ± 0.9, 2.3 ± 0.8) was observed (Fig. 2e, arrows). No CD34 immunoreactivity was detected. In stem cell transplantation group, significant increase in OV-6 immunoreactivity was observed (4.9 ± 1.4, 4.8 ± 1.2) (Fig. 3) with oval cells forming more reactive ductules (ductular-like oval cells) (Fig. 2f, arrows) or appearing as small hepatocyte-like cells. CD34 (antihuman) immunoreactivity reveals presence of positively stained cells (Fig. 2g, arrows) which demonstrates oval cells derived from human origin (hUCB-derived cells). CD34 oval cell score and frequency of exogenous cells incorporated into new ducts were 3.2 ± 1.2 and 2.2 ± 0.9, respectively. Calculation of the acquired data (oval cells +ve for OV-6, antihuman/anti-rat Ab - CD34 +ve cells for antihuman Ab = oval cells of only rat origin). No significant difference was found in the acquired data as compared to the response observed in the liver injury and hepatocyte block group. Previously calculated data reveals that hUCB stem cell transplantation has no effect on endogenous response of oval cells to liver injury.

## Discussion

The liver can regenerate itself by increasing the rate of hepatocyte mitosis and stem cell differentiation into hepatocytes or cholangiocytes. Stem cells are the principle cell lineage for liver regeneration. However, the exact location of these cells is not yet clear [8].

Oval cells represent the progeny of liver stem cells and function as an amplification compartment for the generation of new hepatocytes [9]. This compartment, consisting of small ovoid cells with scant lightly basophilic cytoplasm, is widely used to describe liver progenitors [10]. Here, we describe whether application of stem cells (hUCB) interferes with the natural response of oval cells to injury (represented by the percentage of oval cells in liver tissue and the frequency of new duct formation) or not.

The general principle underlying oval cell activation is based on a combination of liver injury with inability of hepatocytes to proliferate in response to damage [11]. According to these data, the 2-AAF/CCL4 protocol was used in the current work to induce an oval cell response. CCL4 is a known chemical to induce injury in the hepatic centri-lobular area while 2-AAF has been shown to block proliferation of hepatocytes, thus allowing oval cells to continue to proliferate in large numbers. Oval cells lack the ability to convert the 2-AAF to its toxic metabolite, thus escaping its inhibitory effect. This procedure induces a robust response of oval cells reaching its peak 9 days post CCL4 injury, and this was the time determined to detect for oval cell response in our model [12].

Liver injury is followed by extensive changes in variety of enzyme activities as part of the regenerative process [13] (Table 1). In the current work, the ALT, AST, albumin, and bilirubin levels were estimated in different groups 9 days post injury and compared to the control. ALT and AST were significantly elevated in the liver injury and stem cell transplantation groups (*P* value <0.05). The most likely explanation for this transaminase elevation is the release of enzymes from damaged liver parenchymal cells. In contrast, the albumin and bilirubin levels were not significantly different from the control level and this was concomitant with the findings stated by Shakoori and his associates who failed to see any effect on the total protein and bilirubin levels during the first 20 days post injury [14]. The explanation for this may be due to restoration of liver mass and function starting the second week post injury.

In the current work, hUCB was used as a source of hepatic progenitors and this was based on findings noted by Newsome and his colleagues who stated that human umbilical cord blood (hUCB)-derived cells could differentiate into hepatocytes in vivo after their transplantation in immunodeficient mice [5]. This was further modified by Tang and his colleagues who showed that number of positive human AFP and ALB cells in the HUCBSC-treated animals with or without cyclophosphamide did not differ significantly, denoting that immunosuppression had either a mild or no effect on stem cell differentiation in rats [15]. Besides, undifferentiated cells were used in the current work, based on findings of Peters and his colleagues who demonstrated that better results are obtained with undifferentiated stem cells because these cells have very low apoptotic activity responsible for their longer survival. They are lowest in expressing apoptotic proteins, Asp, annexin-V, bax, bad, and bak [16]. These cells derived from the cord blood exhibit higher plasticity than the respective mouse or rat cells [17]. Cell fusion has been implicated as the mechanism by which human cells are seen in the recipient’s liver in most cases [18].

Immunohistochemical analysis of oval cell response in all experimental groups has been evaluated (oval cell score and frequency of cells incorporated into new ducts) and revealed highly significant deviation in stem cell transplantation group (4.9 ± 1.4, 4.8 ± 1.2) as compared to the liver injury (2.4 ± 0.9, 2.3 ± 0.8) and control groups (0.85 ± 0.4, 0.78 ± 0.3) (Fig. 3). OV-6 immunoreactivity was only limited to intraportal bile ducts and terminal duct cells with no evident induced oval cell response in the control group. In the liver injury group, response was significantly increased due to 2-AAF-induced mito-inhibitory effect on liver resident hepatocytes and CCL4-induced oval cell activation. Highly significant deviation was found in the stem cell transplantation group, as OV-6 immunoreactivity was directed to cells of both human and rat origin (using antihuman/rat OV-6 antibody). For mere localization of cells with human origin, CD34 antihuman immunoreactivity was performed. Co-staining of OV-6 and CD34 was technically not possible in liver tissue sections as both antigens are membranously expressed to the same extent [19], and this was treated by staining for OV-6 antigen followed by CD34 in the serial parallel liver tissue section, and co-localization of both antigens was done. Analysis of results reveals no significant change in OV-6 immunoreactivity following stem cell transplantation as compared to the liver injury group.

The application of stem cells in liver therapies seems to be a promising feature for treatment of liver diseases. However, several issues still have to be addressed to fulfill this promise. The fundamental molecular pathways involved in differentiation of hepatocytes and cholangiocytes from stem/progenitor cells need to be explored in more details [20]. Much less is known about the mechanisms of oval cell replication and differentiation [21], and whether the natural response of oval cells to injury is enhanced or decreased by extrahepatic sources of stem cells (like hUCB) is not recently declared. To our knowledge, this is the first time to use an immunohistochemical-based assay to prospectively detect the effect of hUCB stem cells on the natural response of oval cells to liver injury.

## Conclusions

Liver regeneration is a well-organized process by hepatocytes and non-parenchymal cells. Oval cells, resident liver progenitors, play a major role in the natural response of the liver to injury. Extrahepatic stem cells (like hUCB) have recently been used as sources of transplantable hepatic progenitors. In the current work, we aimed to detect the effect of stem cell transplantation (hUCB-derived cells) on oval cell response to injury (represented by oval cell score and the frequency of cells incorporated into new ducts). Based on our results, oval cell response to liver injury is not affected by extrahepatic stem cell transplantation. Thus, in vivo differentiation of cord blood stem cells to liver progenitors or hepatocytes (mostly occurring by cell fusion with resident cells) does not interfere with the natural response of oval cells to injury. These molecular pathways for cellular differentiation and signalling need to be further explored.

## Abbreviations

2-AAF: Acetylaminofluorene
CCL4: Carbon tetrachloride
DMEM: Dulbecco’s modified Eagle’s medium
DMSO: Dimethyl sulfoxide
FBS: Fetal bovine serum
HBSS: Hank’s balanced salt solution
HPCs: Hepatic progenitor cells
hUCB: Human umbilical cord blood
MNCs: Mononuclear cells
